# Meeting Report on Experimental Approaches to Evolution and Ecology Using Yeast and Other Model Systems

**DOI:** 10.1534/g3.115.018614

**Published:** 2015-06-09

**Authors:** Audrey P. Gasch, Gaël Yvert

**Affiliations:** * Laboratory of Genetics and Genome Center of Wisconsin, University of Wisconsin-Madison, Madison, Wisconsin 53706; † Laboratoire de Biologie Moléculaire de la Cellule, CNRS and Ecole Normale Supérieure de Lyon, Université de Lyon, 69007 France

**Keywords:** evolution, yeast, epistasis, genetic architecture

Fall colors and seasonably warm European weather welcomed the third EMBO-sponsored conference on **Experimental Approaches to Evolution and Ecology Using Yeast and Other Model Systems**, hosted at EMBL in Heidelberg, Germany, October 12–15, 2014. Organized by Lars Steinmetz (EMBL Heidelberg and Stanford University) and Michael Knop (University of Heidelberg), this gathering of ∼150 international researchers focused on diverse subjects related to phenotypic evolution and the mechanisms that give rise to it. The meeting included exceptional talks by leaders in the field and new investigators alike that synthesized concepts spanning microevolution in experimental settings to macroevolution across clades and kingdoms. These themes also persisted through the poster presentations and provoked many discussions over dinner and afterward. The impact of the meeting was echoed throughout the week among the participants, several of whom said it ranked among the best meetings they had ever attended.

The talks covered a wide array of evolutionary and ecological systems, from yeast experimental evolution to *Drosophila* genetics, phytoplankton phylogenomics, and microbial interactions in a mammalian host. Even within the diverse range of experimental systems, clear themes emerged from the talks.

## Aneuploidy: a powerful mode of phenotypic evolution

One clear theme of the meeting was the prevalence of aneuploidy in phenotypic evolution. Aneuploidy is a frequent occurrence in pathogenic isolates of *Candida albicans* isolated from human hosts. Judy Berman (Tel Aviv University) discussed how aneuploidy arises after drug treatment via “trimera” cells that form multi-spindle tetraploid intermediates that then missegregate to form aneuploid cells. The process shares several features with aneuploidy generation in mammalian systems. Aneuploidy was strikingly emergent in *C. albicans* mutants selected for mouse colonization in experiments relayed by Norman Pavelka (Singapore Immunology Network).

Like aneuploidy, large-scale segmental duplication is frequently selected in experimental evolution of budding yeast. Maitreya Dunham (University of Washington) presented a clever genetic approach leveraging the *Saccharomyces cerevisiae* deletion collection to map the causal genes within long-known segmental duplications that emerge from nutrient-limitation experiments. The approach clearly demonstrated the additive effects of amplifying linked genes on the same chromosomal arm, providing one hypothesis for the complex benefits afforded by segmental and chromosomal amplification.

The prevalence of aneuploidy in wild isolates of *S. cerevisiae* is becoming clear. Aimee Dudley (Pacific Northwest Diabetes Research Institute) presented several vignettes, including the phenotypic switch in colony morphology caused by changes in chromosomal copy number and the prevalence of aneuploidy and polyploidy emerging in coffee and cacao-associated yeast strains being sequenced in her laboratory. One explanation for the prevalence of aneuploidy in natural strains—up to 30% from a study in the Gasch Laboratory (University of Wisconsin)—may be a mode of dosage compensation discovered in yeast strains, which buffers cells against copy-number variation and aneuploidy until new phenotypes emerge. These results fit nicely with a short talk and poster from Giulia Rancati’s laboratory (Institute of Medical Biology, Singapore), demonstrating that *S. cerevisiae* can readily adapt through chromosome copy number changes to deletion of nominally essential genes.

## Evolutionary trajectories

Despite the recurring emergence of aneuploidy in a variety of natural and experimental evolutions, the absence of detectible aneuploidy in other systems was notable. Andrew Murray (Harvard University) presented work exploring the selection of yeast mutants that overcome near-lethal mutation of the *BEM1* gene. In this case, aneuploidy was not recovered in the selected strains. Rather, the evolutionary trajectory required mutation of other genes in the cytokinesis pathway—but in precise order according to the fitness landscape of the mutations and allele combinations. Murray raised the issue that differences in the frequency and potency of different types of sequence variants will influence the fitness costs and benefits of different mutations under different settings.

Others in the represented laboratories used experimental evolution to track the genetic paths of adaptation. Various flavors of experimental setups were presented, all powered by the recent advances in deep sequencing. In addition to Murray, Greg Lang (Lehigh University) and David Gresham (New York University) showed that whole-genome sequencing of evolving populations can reveal the sequential appearance of beneficial mutations, with frequent competition between interfering clones.

A powerful innovation is the use of barcode tagging, applied either to a set of systematic mutants such as presented by Grzegorz Kudla (University of Edinburgh) or to millions of wild-type cells, as presented by Sasha Levy and Gavin Sherlock (Stanford University). This allowed lineage tracking at unprecedented depth, revealing early dynamics of competition and catalogs of sequential adaptive mutations.

## Epistasis

Several talks covering unrelated studies highlighted the predominant role of epistasis in shaping evolutionary trajectories. Nobuhiko Tokuriki (University of British Columbia) showed that experimental evolution of enzymatic activities is phenotypically revocable but genetically irreversible, because of incompatible mutations.

Sergey Kryazhimskiy (Harvard University) compared evolutionary trajectories starting from closely related yet diversified founder yeast clones. He reported that all founders randomly sampled adaptive mutations from a common pool. Yet fitter founders adapted significantly slower than less-fit founders because of diminishing returns epistasis among beneficial mutations. This resulted in predictable fitness trajectories, despite high degree of randomness at the genetic level.

Eric Alani (Cornell University) spoke of an example of negative epistasis between alleles of mismatch repair proteins segregating among *S. cerevisiae* strains. More specifically, he asked why variants that can promote the accumulation of harmful mutations remain in the population. This was examined by applying salt stress to yeast strains bearing mutagenic and nonmutagenic mismatch repair genotypes. Based on these studies he proposed that the mutagenic state, which can be reversed by mating, allows for rapid adaptation in changing environments.

Epistasis also dominated the properties of the *Drosophila* Genetic Reference Panel (DGRP) reported by Trudy Mackay (North Carolina State University). In a striking example, she cited work in which six mutations were combined in a multi-way cross and produced phenotypic variation in which the additive model had almost no predictive power. This led to active discussions on whether epistasis has been underestimated in less tractable systems, including human GWAS.

Epistasis was not restricted to quantitative genetics; it was also mentioned in talks covering larger evolutionary distances. Jun-Yi Leu (Academia Sinica, Taïwan) reported the rapid co-evolution of essential genes within large multi-subunits complexes, which stabilizes protein–protein interactions and explains incompatibilities that are seen between orthologs of closely related taxa.

Protein–protein interactions were also highlighted by Sandy Johnson (UCSF), who described how alteration of a transcription factor’s interaction with other proteins, through only a few amino acid changes in the factor, can facilitate the evolutionary rewiring of transcriptional networks over millions of years of evolution. This idea was expanded on by Isabel Nocedal from the Johnson laboratory, who showed that evolution of the Ndt80 transcriptional network involved the loss of hundreds of downstream Ndt80 targets and the gain of new gene–target connections as Ndt80 evolved from its ancestral role in meiosis to its derived role in *C. albicans* biofilm formation. Although the audience was familiar with the importance and complexity of genetic and physical interactions, these talks underscored the amazing role these interactions play in short-term and long-term evolution.

## Selection at the level of protein translation and folding

Another theme that grew progressively during the conference was the implication of translation efficiency in adaptive mechanisms. Lars Steinmetz (EMBL Heidelberg and Stanford University) first implicated the potential for translational effects by showing the vast diversity of transcript isoforms for yeast genes, which, on average, encode more than 26 isoforms with distinct boundaries.

Hunter Fraser (Stanford University) reported allele-specific translation rates in yeast hybrids that could compensate or reinforce allelic differences in mRNA levels. He also described a method for correcting sequence biases in ribosomal profiling experiments, which revealed that proline codons constitute pause sites of translation.

Yitzhak Pilpel (Weizmann Institute) presented a study illustrating the evolutionary flexibility of translation coding. Fitness of a yeast strain lacking a specific tRNA from the pool could be restored by experimental evolution via mutations altering anti-codons of other tRNA genes. He also presented comparative genomics evidence that anti-codon evolution may occur frequently in nature. He concluded by discussing different translation programs used by distinct functional categories of genes. In multi-cellular organisms, genes related to development and multi-cellular processes use codons matching tRNAs preferentially expressed in the differentiated tissues, whereas genes involved in proliferation use other codons corresponding to tRNA that are overexpressed in several cancers.

The adaptive role of translation was also reported by Sebastian Leidel (Max Planck Institute for Molecular Biomedicine), who described that yeast deficient in tRNA wobble modification displayed codon-specific slowdown, sensitivity to stress, and increased protein misfolding.

Several other speakers investigated how the efficiency of protein folding modifies adaptation and diversification. Dan Jarosz (Stanford University) reported that transient overexpression of some nonamyloid prion-like proteins can generate phenotypic diversity in the long-term. Kevin Verstrepen (KU Leuven) presented experiments in which polyglutamine stretches in transcription factors generated variation in gene-target activation. This potentially diversifying mechanism can be modulated by chaperones and could have arisen by selection. Protein folding also emerged as a concluding point of Dan Hartl (Harvard University), who addressed the conundrum of why strong signatures of positive selection are often found for ancient proteins whose enzymatic activities have long been optimized. He proposed that cycles of deleterious and stabilizing mutations may be acting on protein folding and stability, rather than enzymatic activity.

## Evolution of genome structure

One particularly interesting hallmark of the conference was to integrate yeast experimentalists with scientists working on different systems, such as bacteria, plankton, flies, and even digital organisms. This integration allowed a discussion of shared interests between diverse scientific communities. Consider the complementarity between several talks describing the evolution of genome structure, whether in nature (Gilles Fischer from CNRS-Université Pierre et Marie Curie studied genome evolution in *Lachancea* yeasts with powerful new bioinformatics tools inferring ancestral genome structures from synteny relationship between extant genomes) or in a synthetic system (Joel Bader from John Hopkins University showed that the artificial "SCRaMbLE" recombination system of the *S. cerevisiae 2.0* genome synthesis project generated complex chromosomal rearrangements and amplifications) or *in silico* (Guillaume Beslon from INSA-Lyon presented artificial evolution of digital microorganisms and how such simulations can help when exploring the role of mutation rate, population size, or lifestyle).

The evolution of genome structure was also investigated at the three-dimensional scale. The laboratory of Romain Koszul (Institut Pasteur) developed a method that exploits chromosomal conformation capture data recovered from 11 species mixed together to re-assemble their genomes *de novo*, generate their genome-wide contact maps, and compare their 3D organization.

## The importance of ecological interactions

If some of the young attendees initially considered yeast and other systems as laboratory-confined model organisms, the conference surely radically changed their perception. In addition to presenting new prion protein classes, Dan Jarosz reported that a signal emitted by bacterial cells could help them survive wine fermentations by inducing [GAR+] yeast prions to thereby attenuate alcohol production.

Norman Pavelka showed that metabolites produced by host microbiota in the mouse gut inhibit *C. albicans* colonization. A combination of mutant screens and in-mice passages identified genetic pathways regulating this interaction, revealing how poor dietary choices might facilitate fungal infections. Diversification into subtypes and interactions between them was also mentioned.

By applying antibiotics to stationary phase bacteria, Joran Michiels (KU Leuven) described the evolution of phenotypic switching, with rates matching the frequency of antibiotic exposures.

Another type of switch emerged in experiments relayed by Paul Rainey (New Zealand Institute for Advanced Study), where selection shifted from acting on selfish cheaters to multi-cellular cohorts. A collective (or multi-cellular) type of *Pseudomonas fluorescens* could generate cheating-but-beneficial bacterial cells via a genetic switch, providing an experimental illustration of the emergence of a multi-cellular life cycle.

Complex interactions during microbial laboratory evolutions, including those driving ecological divergence and incipient speciation in a long-term experimental population of *E. coli* (presented by Zachary Blount, Michigan State University) and the emergence of semi-stable subpopulations in yeast evolution experiments (from Michael Desai, Harvard University) provided additional insight on this topic. Insects were also cited for ecological interactions.

Kevin Verstrepen showed that *S. cerevisiae* can attract *Drosophila* flies by synthesizing volatile esters (flavors desired for beer and wine manufacturing), with a cost to proliferation that is likely compensated by the ecological advantage of recruiting insects as disseminating vectors.

Consistently, Irene Stefanini (Fondazione Edmund Mach, Italy) showed that wasps are major transporters of yeast and that the microenvironment of the wasp gut favors meiosis, revealing how yeast outcrossing may be frequent in the wild.

## Sex, meiosis, and speciation

The principles mentioned above were also evident in presentations covering longer evolutionary time frames. Joe Heitman (Duke University) gave a synopsis on mating system evolution spanning the Tree of Life, highlighting the work of Kevin Roach that demonstrates the potential of a unisex lifestyle to avoid a Muller’s ratchet situation.

Patricia Pukkila (University of North Carolina at Chapel Hill) presented work dissecting hot spots and cold spots of meiotic recombination in the Basidiomycete *Coprinopsis cinerea*. Genomic sequencing of meiotic products identified hot spots of recombination near telomere ends, where transposable elements, paralogs, and sites of CpG methylation are common. The nonrandom distribution of these features may contribute to the uneven rates of sequence evolution across the *C. cinerea* genome.

The role of phenotypic and genetic differentiation was also highlighted by John Taylor (University of California, Berkeley). To identify genetic islands implicated in population differentiation and the phenotypic differences they underlie, he presented several examples of population genomics and “reverse ecology” across fungal species.

Combined, the talks and posters at the meeting provided an overarching view of the evolutionary principles at work in nature, in the clinic and laboratory, and even in industry. Many participants left stimulated by the thought-provoking presentations and interactions, and they expressed hope that the meeting will continue in future years.

